# Recellularization of Native Tissue Derived Acellular Scaffolds with Mesenchymal Stem Cells

**DOI:** 10.3390/cells10071787

**Published:** 2021-07-15

**Authors:** Ebtehal Ahmed, Tarek Saleh, Meifeng Xu

**Affiliations:** 1 Department of Pathology, Faculty of Veterinary Medicine, Assiut University, Assiut 71515, Egypt; ebtehalabdelkhalik@aun.edu.eg; 2 Department of Animal Surgery, Faculty of Veterinary Medicine, Assiut University, Assiut 71515, Egypt; tarek.saleh@vet.au.edu.eg; 3 Department of Pathology and Laboratory Medicine, University of Cincinnati Medical Center, Cincinnati, OH 45267, USA

**Keywords:** organ engineering, mesenchymal stem cells, decellularization, recellularization

## Abstract

The functionalization of decellularized scaffolds is still challenging because of the recellularization-related limitations, including the finding of the most optimal kind of cell(s) and the best way to control their distribution within the scaffolds to generate native mimicking tissues. That is why researchers have been encouraged to study stem cells, in particular, mesenchymal stem cells (MSCs), as alternative cells to repopulate and functionalize the scaffolds properly. MSCs could be obtained from various sources and have therapeutic effects on a wide range of inflammatory/degenerative diseases. Therefore, in this mini-review, we will discuss the benefits using of MSCs for recellularization, the factors affecting their efficiency, and the drawbacks that may need to be overcome to generate bioengineered transplantable organs.

## 1. Introduction

Organ engineering is a novel approach for developing fully or partially functional organs or tissues and capable of compensating for the failure or dysfunction of a specific organ and provides a promising solution to the critical worldwide shortage of organs for transplantation [1]. It is well known that cell therapy has gained significant interest for researchers as a potential new therapeutic strategy for many diseases. However, cell therapy faces several challenges associated with cell availability, survival, engraftment, and differentiation. Moreover, it has been reported that pigs transplanted with stem cells into the infarcted myocardium, experienced more frequent monomorphic ventricular tachycardia, compared to the vehicle-treated group [2]. It has also been reported that cell transplantation may fail to improve the long-term efficacy and increase the incidence of hepatocellular carcinoma for decompensated liver cirrhosis [3]. Acellular scaffolds offer a relatively safe and potentially off-the-shelf solution to cell-based therapies. Combination of cell transplantation with acellular scaffolds will develop substitute organ/tissues and promote endogenous regeneration to save patients suffering from end-stage organ failure [4]. Organ engineering has successfully integrated a functional tissue-engineered cardiac muscle graft to improve myocardial function [5,6,7]. Similar efforts have also been reported in other organs, such as the liver, kidney, lung, pancreas [8,9,10,11,12,13,14]. It is recently reported that a cartilage like tissue has been successfully engineered using human decellularized extracellular matrix (hECM) scaffolds seeded with human adipose stem cells (hASCs) [15]. In addition, a retrospective review evaluates decellularized porcine small intestinal submucosa extracellular matrix (SIS-ECM) used for pericardial closure to reconstruct congenital heart defects on 40 patients aged 2 days to 13 years [16]. No death, or pericardial effusions, or intracardiac/intravascular thromboses occurred related to the SIS-ECM during follow-up 7.85 months (0.5–24 months). The explanted tissue was replaced with organized collagen, and re-endothelialization [16]. Wan et al. [17] used stem cell-seeded human heart valve-derived scaffold (hHVS) to patch infarcted heart, resulting in significantly improving cardiac function and reducing infarct size in a murine model of myocardial infarction. These observations provide the first clinically relevant evidence and models for translating the recellularized native derived-acellular scaffolds into clinical strategies.

The generation of functional bioengineered organs is very complicated. The procedure comprises two important steps. First, a naturally derived acellular scaffold must be prepared from animal or human tissues by removing all cells (decellularization) [18]. Porcine and human organs are often considered good viable sources for generating bioengineered organs. Through using these scaffolds, many promising acellular scaffolds have been developed [19,20,21]. Optimizing the initial step of decellularization is considered to be crucial for the creation of a naturally derived well preserved three-dimensional extracellular matrix (ECM) that provides the functional support needed for cell growth [22]. Second, these acellular scaffolds need to be recellularized, which is the most critical step for the functionalization of these scaffolds. Recellularization is defined as the repopulation of acellular ECM scaffolds with specific cell types. Each scaffold requires specific cell types to be functional and transplantable based on the specific function of the organ under consideration [23]. Myriad cell types such as primary cells [24], cell lines [25], embryonic stem cells [26], adult-derived stem cells [27], and progenitor cells derived from induced pluripotent stem cells (iPSCs) [28] have been studied to repopulate scaffolds. However, none of them is considered an ideal cell source due to imperfections in each cell type and lack of long-term in vivo organ transplantation studies [29]. The optimization of the most appropriate cells is still challenging. All of the cells are needed to be tested extensively to increase their functionality and overcome their limitation. Mesenchymal stem cells (MSCs) are one of the widely studied cell types. MSCs have shown a remarkable potential of repopulating various acellular scaffolds, making it a kind of promising functional cell type [30]. MSCs are multipotent stromal cells that possess a self-renewal and differentiation capacity [31,32]. They are easily obtained from multiple tissue sources such as bone marrow, adipose tissue, placenta, umbilical cord, etc. [33]. More importantly, MSCs have been used for treating various diseases as it has been widely reported that they promote organ integrity due to their immunomodulatory, antifibrotic, angiogenic, antiapoptotic, and mitotic properties [34]. MSCs can migrate to injured areas, differentiate into tissue-specific cells, and replace injured cells while, at the same time, reducing inflammatory cytokines [35,36]. Additionally, they significantly enhanced angiogenesis and neovascularization through directly trans-differentiating into blood vessel phenotypes and via releasing paracrine factors [37,38,39]. Recently, MSC-derived extracellular vesicles have been studied in some acute and chronic tissue injuries, and the results proved that these microvesicles may serve as a potential innovative treatment strategy to overcome the limitation of conventional cell therapy [40,41,42,43].

Based on the findings of the preclinical studies, the generation of fully functional recellularized transplantable organs is still challenging. Many aspects need to be further optimized in terms of decellularization, modification of the scaffolds to produce structurally and biochemically preserved acellular tissue-derived ECMs, and consequent repopulation with different types of tissue-specific cells. Some of the studies mentioned in this review provided a promising result that confirms the feasibility of using these repopulated scaffolds in damaged organ replacement. In this mini-review, we mainly discuss the promises and limitations of using MSCs for the recellularization of native tissue (organ) derived acellular scaffolds.

## 2. Role of MSCs in the Natural-Derived Scaffolds Mediated Regeneration

MSC binding within scaffolds occurs by the interaction of specific cellular integrins with different ECM proteins [44]. MSCs may attach to specific regions within the decellularized scaffolds, especially those consisting of abundant collagen I and IV, laminin, and fibronectin [45]. These interactions may, in turn, regulate the behavior of MSCs and allow the seeded MSCs to acquire characteristics of native cells present in the scaffolds before decellularization. MSCs can respond appropriately in terms of cell shape, differentiation, proliferation, and migration to the different ECM compositions and cell niches accordingly [46]. Thus, MSCs could play a critical role in using scaffolds to mediate organ and/or tissue regeneration, including differentiation of functional cells, regulation of immune, and increase of angiogenesis.

### 2.1. Differentiation of Functional Cells

Decellularized ECM materials contain a unique composition of bioactive molecules that can guide MSCs to differentiate into multiple tissue-specific lineages [47]. MSC behavior, multiplication, and fate substantially controlled by the mixture of ECM proteins and growth factors comprised by the ECM, where they grow [48]. One of the ECM components, collagen II, promotes MSC chondrogenic differentiation, which plays a vital role in matrix remodeling. This chondrogenic differentiation was observed when MSCs were grown on collagen II hydrogel [49], while ECM protein collagen VI greatly enhanced MSC myogenic differentiation after muscle injury [50]. In addition, fibronectin, fibromodulin, biglycan, and decorin present in tendon ECM induce tenogenic differentiation of seeded MSCs [51]. MSCs can respond according to the mechanical properties and signals of the scaffolds by a mechano-transduction process. For example, rigid scaffolds may enhance the osteogenic differentiation of MSCs, while pliable ECM materials may favor their adipogenic differentiation [48,52]. A study that investigated the osteogenic potential of MSCs isolated from umbilical cord Wharton’s jelly (UC-MSCs) indicates that osteogenic differentiation of UC-MSCs was enhanced on a stiff substrate, compared to soft substrates [53]. These results also show that substrate stiffness can regulate MSC differentiation. ECM collagen fibers alignment also could influence the MSC differentiation, wherein the study of Marinkovic et al. [48] showed that uniformly aligned collagen fibers stimulate MSC osteogenic differentiation, and the irregular aligned collagen fibers stimulate MSC adipogenic differentiation.

Additionally, the differentiation efficiency of MSCs can also be affected by other neighboring cells that have a close contact with MSCs [54]. Co-culturing of MSCs with organ-specific primary cells supports primary cell proliferation and enhances MSC differentiation into tissue-specific cells. MSCs, when co-cultured in vitro with endothelial cells, enhance tube formation and vascularization [55]. This outcome can be induced by direct cell–cell contact or by indirect stimulation from bioactive molecules secreted from neighboring cells [56].

### 2.2. Regulation of Immune Action

Survival and reducing the risk of rejection of a transplanted graft rely mainly on minimizing any elicited host immune response [57]. MSCs possess an immunomodulation effect where they can alter the host immune reaction. Suppression of the host immune reaction against the implants would allow an effective constructive tissue remodeling, delay any biodegradation, and prolong the survival of the transplanted tissue/organ until the recipient make their ECM required for achieving a successful ECM turnover [58]. MSCs seeded in transplanted scaffolds can lead to significant inhibition of leukocyte infiltration which inhibits ECM destruction and injury of the graft postoperation [59]. Macrophage phenotyping is considered to be a good indicator for implanted scaffold biocompatibility [45,60]. The M1 macrophage phenotype produces IL-1β, IL-6, and TNF-α which are recognized proinflammatory cytokines and can lead to graft destruction when acting in concert with Th1 cells [61]. However, the M2 macrophage and Th2 cells have a regenerative tissue response. The implantation of MSCs-recellularized scaffolds could encourage the polarization of macrophages toward constructive phenotype M2 [62]. MSCs suppress the proliferation of T-cells, regulate the ratio of Th1/Th2, control the functions of regulatory T cells (Tregs) [59], and secrete interleukins -10 (IL-10) [63,64]. Additionally, MSCs increase the expression of MMP-1, MMP-3, and MMP-13, and maintain the remodeling process of the ECM [65].

### 2.3. Increase of Angiogenesis

MSCs have been widely studied as potential treatments in preclinical models of stroke, myocardial infarction, and peripheral artery disease because of their unique angiogenic properties [66,67,68]. MSCs play an important role in supporting tissue remodeling and enhancing neovascularization in the decellularized scaffolds [69,70]. Sarig et al. [71] reported that seeding of MSCs after the re-endothelization of decellularized porcine cardiac ventricular ECM (pcECM), with endothelial cells leading to a patent vascular network and vascular maturation. MSCs can enhance the survival and proliferation of endothelial cells through secreting paracrine factors, which ensure blood vessels with adequate endothelial coverage to prevent thrombosis [72]. Growth factors secreted from MSCs enhance the proliferation and infiltration of surrounding host cells within the scaffolds and stimulate angiogenesis in the implanted area [55,73]. Secreted VEGF, HGF, and bFGF influence the migration of host endothelial progenitor cells and differentiation of these cells into endothelial cells, resulting in significantly increased vascular sprouting and graft regeneration [74,75]. MSCs are also reported to mediate angiogenesis through activating the angiopoietin 1 (Ang1)–Tie 2 signaling pathway. The activation and phosphorylation of Tie2 stabilize the neovasculature by enhancing peri-endothelial cell recruitment [76,77]. Furthermore, MSCs can migrate through the wall of the blood vessel to reach the tunica adventitia and differentiate into pericytes to provide the vascular tissue the required integrity to hinder the risk of hemorrhage [12]. Similar results were obtained in the study by Wang et al. [78], where MSCs were successfully used in the recellularization of acellular myocardial scaffolds, in which the seeded cells expressed von Willebrand factor (vWF), an endothelial marker, within the vascular space. The angiogenic effect of MSCs allows the implanted scaffolds to receive nutrients required for tissue remodeling. 

**Figure 1:**
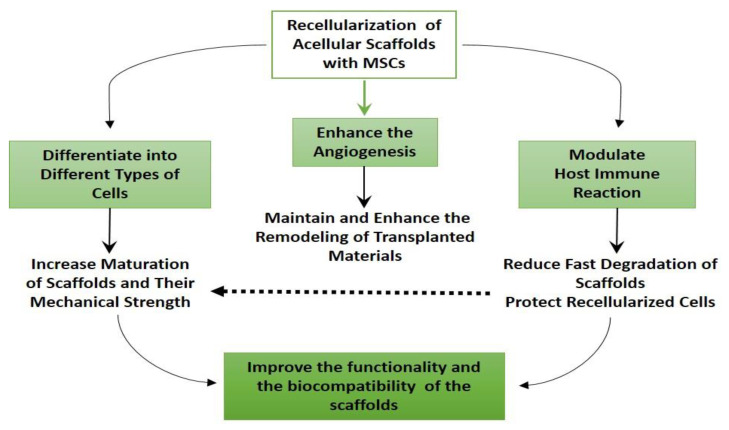
The roles of MSCs in scaffoldmediated regeneration.

The roles of MSCs in scaffold mediated regeneration are summarized in Figure 1.

## 3. Recellularization of Scaffolds with MSCs

### 3.1. Methods of Recellularization

**Figure 2:**
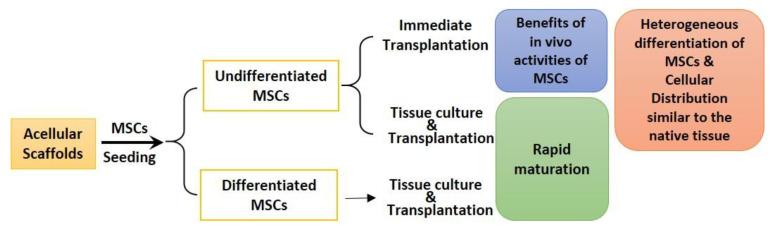
Potential outcomes of different recellularization strategies using MSCs.

Based on the differentiation state of MSCs, three strategies may be used to recellularize acellular scaffolds (Figure 2).

The first strategy is to inject or place MSCs in an undifferentiated state and perform in vitro culture to allow cell attachment and proliferation [79]. Consequently, these cultured cells are transplanted for further in vivo differentiation and maturation [79]. The advantage of this strategy is to shorten the time needed for the seeded cells to attain their full maturation and function after in vivo implantation. However, this in vitro expansion and culture may subject the implanted cells to hypoxia. In addition, these cells may lose some of their beneficial anti-inflammatory or immunomodulatory functions [80]. 

The second strategy is to inject undifferentiated MSCs into decellularized scaffolds immediately before transplantation. In this method, the in vitro tissue culture step is skipped. The in situ cell seeding has the advantage of enhancing cell differentiation and distribution, and protecting the cells from the deleterious effects associated with the pre-seeding step [59].

The third strategy involves seeding the decellularized scaffolds with already differentiated MSCs, followed by performing in vitro organ culturing before transplantation [81]. This recellularization strategy provides complex scaffolds with tissue-specific phenotypes [23]. To date using multiple cell types for proper repopulation is challenging especially in dense parenchymatous organs such as the liver and kidney [82].

### 3.2. The Factors That Are Responsible for Recellularization Efficacy 

The recellularization of an acellular scaffold is mainly dependent on the state of the scaffold and MSC properties. Table 1 lists the important factors that could affect the success of the recellularization applications. These factors include the status of scaffold source, decellularization protocol, recellularization strategy, the cell type and number, etc. 

#### 3.2.1. The Anatomical Structure and Pathological Conditions of Scaffold Sources

The age and health status of the native tissue before recellularization affect the structural and biochemical properties of the generated decellularized tissues. Sokocevis et al. [87] reported that lungs obtained from the aged patients could be used for proper decellularization and subsequent recellularization with MSCs. However, it is found that the colony-forming cells of MSCs cultured in ECM obtained from old mice (old-ECM) were marginally lower than that cultured in ECM from young mice (young-ECM) [92]. The anatomical barriers within the scaffolds and the density of complex organ tissues can also greatly influence the invasion and distribution of MSCs [23,93]. The cellularity in the homogeneous thinner tissues may be higher than that of heterogenous thicker tissue [94]. In addition, the risk of hypoxia and further cell necrosis are higher in dense tissue recellularization [89]. On the other hand, fibrosis of tissue increases ECM stiffness due to the accumulation of ECM proteins and dysregulation of some ECM components, which may, in turn, affects the proper distribution, viability, differentiation of the recellularized cells [95]. Wagner et al. [96] and Sokocevic et al. [87] compared acellular lung scaffolds from cadaveric patients with the chronic obstructive pulmonary disease to scaffolds obtained from normal healthy lungs. The results indicate that the emphysematous acellular scaffolds were not appropriate for recellularization and transplantation, since the disrupted ECMs could not support the growth of newly added cells for very long. Therefore, recellularization strategies for each tissue type require optimization to generate a functional bioengineered organ.

#### 3.2.2. Method of Decellularization

Decellularization approaches remove the cellular component and attenuate the immunogenicity of the transplanted scaffolds. The structural architecture and the biochemical components retained in the scaffolds after decellularization are varied according to the detergent used and decellularization protocol. Katsimpoulas et al. [97] showed that decellularized aortas elicited minimal immune response and lymphocytic infiltration compared to the native allografts. Moreover, Hundepool et al. [98] further reported that optimizing the decellularization protocol is crucial to reducing cellular debris and immunogenicity without affecting tissue architecture. The combinations of different decellularizing protocols have been reported to overcome immune rejection after transplantation [99]. However, some recent studies indicated that decellularization approaches are insufficient to ensure the elimination of antigenic components, and therefore, this technique needs to be modified to generate immunologically accepted scaffolds [100,101,102]. To achieve adequate cell removal without disrupting the organ native microenvironment, the protocols and the detergents used in decellularization must be carefully optimized.

**Figure 3:**
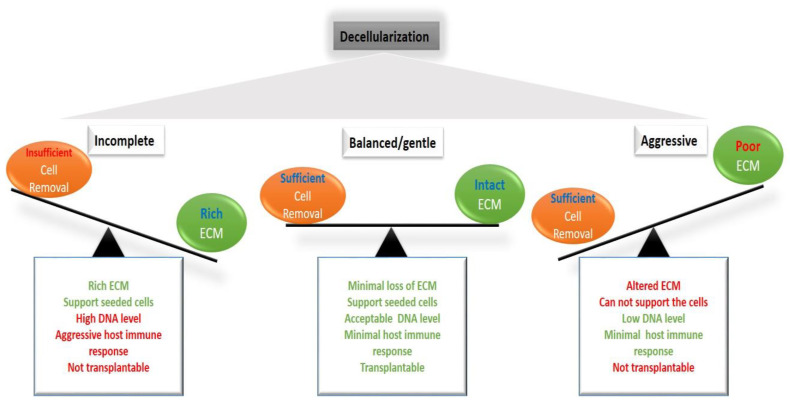
The effect of decellularization on the subsequent organ repopulation and organ transplantation potentials.

The biochemical components of decellularized scaffolds appear to be dictated by the decellularization method [103] since this procedure can alter components of the scaffolds, particularly loss of bioactive molecules [22]. The alteration of the main ECM components—collagen, fibronectin, and laminin—may affect the initial binding, proliferation, and distribution of MSCs [104]. Wallis et al. [85] showed that the MSCs were initially localized in regions rich in fibronectin, collagen I and IV, or laminin in the lung tissues treated with different decellularizing reagents. A Triton-X 100 based decellularized uterus showed more distribution and homogenization of MSCs than did a sodium deoxycholate (SDC)-based-decellularized uterus [79]. Different scaffold stiffness resulting from the use of harsh detergents may also affect cell adhesion and distribution [88,89]. Saha et al. and Leipzig et al. [105,106] demonstrated that the elastic modulus of the scaffolds greatly influences neuronal stem cell differentiation. Moreover, long-term storage of decellularized scaffolds, as well as the type of sterilizing agent used, greatly influences the survival of inoculated MSCs [107]. It is well known that maintaining the native mechanical and biochemical properties of scaffolds after decellularization is necessary to keep normal nutrient diffusion, cell growth, and differentiation [108]. Therefore, optimizing gentle non-destructive decellularizing and sterilizing protocols may retain more essential active ECM components needed for ensuring successful recellularization (Figure 3).

#### 3.2.3. The Surface Modification of Scaffolds

Modification of acellular scaffolds to overcome their limitations in terms of activity, structural stability, and functionality would additionally improve the recellularization efficacy with MSCs. Modification by crosslinking or coating with different improving materials such as heparin, fibronectin, gelatin, peptides, or extracellular matrix particles or nanomaterials are used to enhance the functionality and hemo/biocompatibility of these scaffolds [109,110,111,112]. The combination of acellular porcine aortic value with porous matrix metalloproteinase (MMP) and degradable polyethylene glycol (PEG) hydrogel can mechanically promote bone marrow MSC (BM-MSC) attachment, growth, and differentiation. Consequently, recellularized MSCs promote constructive tissue remodeling by expressing the M2 macrophage phenotype, which could enhance the biocompatibility of transplanted values and inhibit their rapid destruction [113]. Moreover, Wang et al. [69] confirmed that surface modification of the scaffold with gelatin or fibronectin enhances proper MSC attachment and proliferation in cardiac tissue engineering. Dong and his colleagues [114] also examined the effect of conjugating the tri-amino acid sequence, arginine–glycine–aspartate “RGD” polypeptide to bovine pericardium after decellularization and found that RGD peptide enhances the initial attachment of MSCs and improves cellular growth and proliferation, compared to the unmodified scaffolds.

#### 3.2.4. Source and Abundance of MSCs

The most common types of MSCs used in recellularization are obtained from adipose stromal cells (AD-MSCs) and bone marrow (BM-MSCs). Both are accessible sources of MSCs and can be obtained in adequate numbers. These cells have extensive proliferative capabilities to undergo multilineage differentiation. Bonvillain et al. [30] compared the efficiency of two types of cells in acellular lung repopulation and found that both cell types showed the same invasion, attachment, and proliferation capabilities. Moreover, Wang et al. [64] reported that Schwann cells transdifferentiated from either BM-MSCs- or AD-MSCs-enhanced recovery of nerve tissue injury without intergroup differences. Li et al. [115] reported that BM-MSCs possess enhanced osteogenic and chondrogenic differentiation activity and secrete high levels of HGF and VEGF. However, AD-MSCs convey a more potent immunomodulatory effect than BM-MSCs. In addition, compared to BM-MSCs, AD-MSCs were more easily isolated from their respective source tissues.

Cellular density is another factor that should be optimized to ensure successful recellularization. Generally, the number of cells required to be seeded depends on the organ’s structure and functions [23]. For example, to achieve successful transplantation, organs such as the heart need nearly full recellularization [116,117], in contrast to the liver that could be functional with a lesser cellular density [118]. VeDepo et al. [83] examined the difference of high and low MSC density in recellularization of acellular aortic valves and found that high MSC density improved biomechanical properties of both the recellularized tissues and cell phenotypes. Conversely, Tiemann et al. [89], in a study of sheep uterus scaffolds, did not observe a great impact when a higher number of MSCs were seeded. These results elucidate the importance of optimization of the number of cells for the different scaffolds obtained from various tissues.

#### 3.2.5. The Microenvironment of Cell Culture

Culturing environments can influence the distribution and proliferation of MSCs [119]. Crabbé et al. [86] compared dynamic and static MSC culture conditions and indicated that MSCs cultured within the decellularized ECMs under dynamic bioreactor enhance tissue regeneration and remodeling. The dynamic bioreactor might provide cultured cells with enhanced amounts of nutrients and oxygenation, prevent cell clustering, and ensure uniform cell distribution. In addition, the differentiation of MSCs into fibroblast-like cells was enhanced in dynamic conditions, indicating that the recellularized MSCs could efficiently generate their own ECM proteins, which, in turn, supports subsequent ECM remodeling of the transplanted graft [86].

Culturing media could also influence the behaviors of MSCs. Syedain et al. [120] reported that the culturing medium can influence MSC migration and proliferation capacity without affecting their differentiation. In the study of Daly et al. [27], BM-MSCs seeded in mouse lungs through intratracheal inoculation showed different cellular behavior depending on the medium in which MSCs were cultured. MSCs incubated in basal medium (DMEM) were better than those incubated in small airways growth medium (SAGM). Additional supplementation of DMEM with certain elements as ascorbic acid or insulin can also influence MSC distribution, cellular density, as well as differentiation [121].

Seeding approaches need to be properly optimized to ensure equal cell distribution, as direct multiple injection recellularization may result in cell cluster formation as well as ECM damage [122]. On the other hand, vascular cell delivery may lead to cellular aggregation and the blockage of vascular networks [123,124]. 

### 3.3. Limitations of MSCs for Recellularization of Acellular Scaffolds

MSC-recellularized matrices still may suffer various drawbacks regarding the source and isolation procedure, properties, and safety of MSCs.

First, the expansion of autologous MSCs is time consuming, compared to allogeneic MSCs, which could be collected in substantial quantities from healthy donors and stored for use. However, still, the optimal amount of allogeneic MSCs that could be safely transplanted without complications is unknown in large measure due to the potential production of alloantibodies [125,126,127]. The obtaining of perfect quality and quantity of autologous MSCs is limited in some cases. It is difficult to obtain useful numbers of MSCs from the elderly where bone marrow sources may be diminished or in patients lacking sufficient body adipose tissue. Additionally, isolation of MSCs from patients who suffer from systemic diseases that affect the bone marrow or alter the properties of MSCs may reduce the chance of their using for recellularization [92,126,128,129,130,131]. One interesting study innovated a novel technique to detect the cells that maintained a youthful phenotype among the MSCs obtained from the elderly donors and expanded in young peoples’ MSC-derived ECM to obtain large quantities of high-quality MSCs from elders [132]. Sun et al. [92] also reported that the intracellular levels of reactive oxygen species in MSCs obtained from elderly donors cultured in young ECM were reduced 30–50%, compared to those maintained on old ECM or plastic. These results suggested the possibility to rejuvenate MSCs from the elderly population by culture them on a young ECM.

Second, the isolation processes are varied, which consequently affects the quality, homogeneity, and activity of isolated MSCs, making the potential fates too difficult to be predicted [133,134,135]. 

Third, the undifferentiated MSCs could be affected by the surrounding donor tissues/cells. These may alter the process of proper MSC differentiation and lead to the formation of different kinds of unwanted cells within the MSCs population [136]. Fortunately, this issue may be of minor concern due to the protective role of the surrounding ECM of the transplanted scaffold. Additionally, this limitation may not be observed in the case where differentiated MSCs are used. However, using such differentiated cells still requires long-term evaluation in terms of immunogenicity and functionality.

Finally, the safety and long-term potential complications related to the use of MSCs remain untested. It is well known that MSCs can differentiate into different tumor-associated cells or increase the tumorigenicity of neighboring tumor cells in vivo, although mixed reactions are observed against different tumor cells in vitro [134,137,138]. Therefore, MSC therapy would not be recommended for the patients who have or even tend, to have tumors, since it is difficult to completely control the fate of the cells after transplantation.

## 4. Conclusions

Organ engineering seeks to provide fully or partially functional organs or tissues to compensate for the failure or dysfunction of a specific organ. The critical steps for generating a bioengineered organ are the preparation of acellular scaffolds and the recellularization of these scaffolds. The recellularization of acellular scaffolds using MSCs showed promising results but still needs to be thoroughly studied and performed preclinically in different models to optimize all factors and overcome the different potential limitations. 

## Figures and Tables

**Table 1:** The factors that could affect the success of the recellularization applications.

Scaffold Source [Ref.]	Decellularization	Recellularization	Cells (Number)	In Vivo	Main Outputs
Aortic valve-Ovine [83]	Thawing, osmotic shock, TX-100, sodium lauroyl sarcosine, and benzonase	Seeded into valve lumen in the static chamber incubated for 2 wk.	BM-MNCs, BM-MSCs, VIC	-	A high concentration of BM-MSCs showed a good phenotype and improved the mechanical and biochemical characteristics of scaffolds.
Esophagus-Pig [84]	2% SDS, 5 mM EDTA, hypotonic water, DNase	Incubated inside a rotating agitator for 21 d.	BM-MSC (2.5 × 10^5^)	-	Successfully attach and proliferate throughout the acellular esophageal wall.
Lung-Rhesus macaque [30]	TX-100, SDC, NaCl, DNase	Inoculation of cells into the lungs, then and slicing and culturing for 7 d.	Rhesus primary BM-MSC and AD-MSC	-	MSCs obtained from either BM-MSCs or AD-MSCs are suitable to recellularized the lung scaffolds.
Lung-Rat [12]	TX-100, SDS	Injecting of cells through the pulmonary artery and pulmonary vein.	Lung ECs (4 × 10^7^); AD-MSCs (1 × 10^7^)	lung was replaced for 3 h	Differentiation into perivascular cells; Upregulated angiogenic growth factors; Increase ECs proliferation and survival.
Lung-Mouse [85]	Triton/SDC-based SDS-based CHAPS-based	Intratracheal injection.	BM-MSCs; C10 epithelial cells culturing for 1 or 14 days (2 × 10^6^)	-	BM-MSCs localized the regions enriched with FN, Col I, IV, and laminin. No differences in attachment and proliferation among various scaffolds.
Lung-Mouse [86]	TX-100; SDS	Intratracheal injection; then connected to RWV for 10, 24 d.	BM-MSCs EC cell line (4 × 10^6^)	-	BM-MSC growth and differentiation into fibroblast-like cells.
Lung-Mouse [87]	1% TX-100, 2% SDC, 1 M NaCl, DNase	Inoculated to the lung for 30 min, sliced lung, and incubated for 28 d.	MSCs or lung ECs (1 × 10^6^)	-	Binding, proliferation, and viability were not good in the densely fibrotic lung or the emphysematous lung.
Lung-Mouse [27]	0.1% TX-100, 2% SDC, DNase.	Intratracheal inoculation. cultured for 1 month in different media.	BM-MSCs (2 × 10^6^)	-	Attached well in regions enriched with collagen I and IV and laminin. Proliferation well in MSCs basal medium.
Myocardial patches-Rat [88]	AR using ASB-14	Placing of cell and culturing for 7 days.	Human or murine MSCs (1000 cell/mm^2^)	implanted in adult male mice for 12 wk	Inflammatory responses were altered Unexpectedly, AR- MSC intense inflammatory reaction.
Ovary-Mouse [89]	P1: 0.5% SDS P2: 2% SDC P3: P1 + P2.	5 successive injections culturing for 5 days.	BM-MSCs (2 × 10^5^)	-	Cells distributed within scaffolds Good recellularization and proliferation capacity.
Pancreas-Mouse [13]	1% T-100, 0.1% ammonia, DNase 200 U/mL.	Inoculated through the portal vein and cultured for 5 d.	hPL-MSC (6−10 × 10^5^)	implanted to pancreas for 45 days	MSCs differentiation; bioengineered functional pancreas generated.
Pulm. valve conduits-Pig [74]	Tris, EDTA, aprotinin, SDS, Tris NaCl buffer	Injection into the pulmonary arterial wall and the annulus of the pulmonary valve.	autologous BM-MNCs or BM-MSCs	Native pulmonary valves were replaced.	Increasing recolonization; altering the inflammation and structural deterioration; paracrine factors secreted stimulate cells differentiation.
Sciatic nerve- Rat [81]	3% TX-100, 4% SDC	Cells were used to recellularize acellular nerve.	Schwann like-cells (from BM-MSCs, and AD-MSCs) (5 × 10^5^)	Grafting of the left sciatic nerve	Promoting nerve regeneration; Protection against muscle atrophy; Increase sensitivity to stimulus.
Trachea–Pig [59]	P1: TX-100 P2: hypotonic sol + hypertonic sol	Direct transplantation + inoculated on external and luminal surfaces.	MSC TEC	samples collected after 7 and 21 days P. O.	Altering mononuclear cellular infiltration.
Umbilical artery-Human [90]	CHAPS buffer, SDS	Cells were incubated for 96 h.	EPCs from Wharton jelly MSCs		Regenerating injured vascular tissue.
Urinary Bladder–Rat [91]	1% SDS; TX-100; DNase	seeded on both sides of the bladder segment and culturing for 7 d.	BM-MSCs (1 × 10^6^)	Implantation following Partial cystectomy.	Immunomodulatory action Enhanced muscle regeneration and tissue remodeling.
Uterus-Rat [79]	1%TX-100 + 4%DMSO + PBS; 1%TX-100 + 4%DMSO + H_2_O; 2%SDC + H_2_O	Multiple direct injections	Primary uterine cells + BM-MSCs culturing for 3 d (7.3 × 10^6^)	Grafting of intrauterine defects	Modulating the immune response Differentiation into uterine specific cells.

AR = antigen removal; ASB-14 = amidosulfobetaine-14; RWV = rotating wall vessel bioreactor; hPL-MSC = human placenta-derived MSC; PO = post-operation; TEC = Tracheal epithelial cells; EPCs = endothelial progenitor cells; VIC = Valve interstitial cells; FN = fibronectin, Col I, IV = collagen 1, IV; SDS = sodium dodecyl sulfate; TX = triton X-100; BM-MSC = bone-marrow-derived mesenchymal stem cell; AD-MSCs = adipose-derived mesenchymal stem cells; SDC = sodium deoxycholate; EDTA = ethylenediaminetetraacetic acid. Hypotonic sol: Tris-HCl, EDTA, triton-X 100, protease inhibitor; hypertonic sol: Tris-HCl, EDTA, triton-X 100 1%, KCl.

## Data Availability

Data sharing not applicable.
